# A Counseling Mobile App to Reduce the Psychosocial Impact of Human Papillomavirus Testing: Formative Research Using a User-Centered Design Approach in a Low-Middle-Income Setting in Argentina

**DOI:** 10.2196/32610

**Published:** 2022-01-13

**Authors:** Victoria Sanchez Antelo, Lucila Szwarc, Melisa Paolino, Diana Saimovici, Silvia Massaccesi, Kasisomayajula Viswanath, Silvina Arrossi

**Affiliations:** 1 Centro de Estudios de Estado y Sociedad Consejo Nacional de Investigaciones Científicas y Técnicas Ciudad Autónoma de Buenos Aires Argentina; 2 Centro de Estudios de Estado y Sociedad Ciudad Autónoma de Buenos Aires Argentina; 3 Secretaria de Salud de Ituzaingó Instituto Provincial del Cáncer Ministerio de Salud de la Provincia de Buenos Aires Ituzaingo Argentina; 4 McGraw-Patterson Center for Population Sciences Dana-Farber Cancer Institute Boston, MA United States; 5 Department of Social and Behavioral Sciences Harvard T H Chan School of Public Health Harvard University Boston, MA United States

**Keywords:** mHealth, mobile application, counseling, HPV test, cervical cancer, health belief model, integrated behavioral model, patient education, Argentina

## Abstract

**Background:**

Human papillomavirus (HPV) testing detects sexually transmitted infections with oncogenic types of HPV. For many HPV-positive women, this result has negative connotations. It produces anxiety, fear of cancer or death, and disease denial. Face-to-face counseling could present many difficulties in its implementation, but a counseling mobile app could be practical and may help HPV-positive women reduce the psychosocial impact of the result, improve their knowledge of HPV and cervical cancer, and increase adherence to follow-up.

**Objective:**

This study aims to understand HPV-tested women’s perceptions about an app as a tool to receive information and support to reduce the emotional impact of HPV-positive results. We investigated their preferences regarding app design, content, and framing.

**Methods:**

We conducted formative research based on a user-centered design approach. We carried out 29 individual online interviews with HPV-positive women aged 30 years and over and 4 focus groups (FGs) with women through a virtual platform (n=19). We shared a draft of the app's potential screens with a provisional label of the possible content, options menus, draft illustrations, and wording. This allowed us to give women understandable triggers to debate the concepts involved on each screen. The draft content and labels were developed drawing from the health belief model (HBM) and integrative behavioral model (IBM) variables and findings of mobile health literature. We used an FG guide to generate data for the information architecture (ie, how to organize contents into features). We carried out thematic analysis using constructs from the HBM and IBM to identify content preferences and turn them into app features. We used the RQDA package of R software for data processing.

**Results:**

We found that participants required more information regarding the procedures they had received, what HPV-positive means, what the causes of HPV are, and its consequences on their sexuality. The women mentioned fear of the disease and stated they had concerns and misconceptions, such as believing that an HPV-positive result is a synonym for cancer. They accepted the app as a tool to obtain information and to reduce fears related to HPV-positive results. They would use a mobile app under doctor or health authority recommendation. The women did not agree with the draft organization of screens and contents. They believed the app should first offer information about HPV and then provide customized content according to the users’ needs. The app should provide information via videos with experts and testimonies of other HPV-positive women, and they suggested a medical appointment reminder feature. The app should also offer information through illustrations, or infographics, but not pictures or solely text.

**Conclusions:**

Providing information that meets women’s needs and counseling could be a method to reduce fears. A mobile app seems to be an acceptable and suitable tool to help HPV-positive women.

## Introduction

### Background

Worldwide, more than 600,000 new cases and 240,000 deaths occur annually due to cervical cancer (CC), which disproportionately affects socioeconomically vulnerable women [1]. High CC mortality is related to problems across the cancer control continuum, including low screening coverage and loss to follow-up, diagnosis, and treatment [2,3]. In recent decades, human papillomavirus (HPV) testing has been developed as an alternative screening method. HPV testing has become the standard of care and a main strategy to accelerate the elimination of CC [4]. HPV testing detects sexually transmitted infections (STIs) with oncogenic types of HPV. Triage tests are used to identify HPV-positive women who will require diagnosis and treatment. In Argentina, the prevalence of high-risk HPV among screened women is around 13% [5]. HPV-positive women with negative triage will require rescreening in 12/18 months. Thus, infection with an oncogenic type of HPV can be detected, but positivity does not necessarily mean that the infection will cause cancer or that the woman will require treatment.

These particular characteristics of HPV results may have a negative impact on the psychosocial health of tested women [6,7]. As with other STIs, HPV is often accompanied by a host of negative beliefs and may cause fear, stigma, shame, and anxiety [8,9]. HPV-positive women are usually the target of negative stereotyping and may be questioned about their sexual behavior, situations that only increase their psychological burden [10]. HPV-positive results can also produce anxiety, fear of cancer or death, and disease denial [11-13]. Thus, HPV-positive results disrupt women’s lives [14], characterized by uncertainty and ambiguity, and cause emotional impact and changes in their everyday life [15-19].

Relating an abnormal screening test with cancer and inevitable death can result in women being hesitant to continue follow-up procedures [17,19,20]. Women with high distress after abnormal screening tests are more likely to exhibit avoidant rather than adaptive coping strategies [10,21,22], making it more likely for nonadherence to occur [23]. Moreover, stigma related to the screening test result with an STI can also lead to the abandonment of follow-up and treatment [18]. Thus, HPV’s psychosocial impact might not only diminish women’s quality of life but also reduce their ability to complete diagnosis and treatment, which are essential steps in preventing CC. There is a critical need for interventions to reduce the psychosocial impact of HPV positivity and increase women's capacity to adhere to follow-up.

Women’s negative perceptions and concerns related to a positive HPV result decrease when their information needs are met and reassuring information is provided [24,25]. In addition, counseling has been extensively used for a wide range of health problems [26-28] and has been shown to increase HIV-testing rates [29,30], improve adherence to treatments [31], increase the quality of life of patients with cancer, and facilitate informed health decisions [26]. The World Health Organization recommends counseling as a strategy for interpersonal communication between the health care provider and the woman, as it allows women to become more informed and knowledgeable about HPV and CC prevention; offers a space to discuss sensitive topics, such as sexuality, disease, and death; and may encourage them to adopt preventive practices [32].

However, providing person-to-person counseling to all HPV-positive women presents implementation obstacles that may affect its quality. The main limitations are that it involves 1 or even several consultations, where each is time-consuming and should be provided by well-trained providers in consultation rooms that guarantee privacy [33-35]. Studies have pointed out that women often receive limited support and tools to cope with the psychosocial impact of HPV positivity, especially in settings with limited health resources [17,20,36]. In addition, although prevention programs from all countries in Latin America and globally produce information materials, in general this information is often provided without considering the barriers to comprehending complex information related to HPV [37-39].

In Argentina, women have reported problems in comprehending information during result delivery [20,40]. A study showed that provider communication is mainly focused on informing women of follow-up steps, leaving little to no room to address women’s concerns, such as the sexual transmission of the virus and its link with cancer [20]. Results from a study carried out among 200 HPV-positive women showed that almost half of them considered that the information provided by health providers was confusing and lacked clarity [40].

Therefore, women undergoing HPV testing need innovative solutions to provide them with information, counseling, and support that do not depend on extensive use of human resources and time and that may increase their autonomy in accessing patient-centered information. Mobile health (mHealth) interventions can enhance the relationship between patients and health services and have been shown to increase adherence in primary care and gynecology care settings [41-43]. In particular, the use of mobile apps to communicate with patients showed the improvement of health outcomes for a wide range of health conditions, including mental health [44-46]. In cancer care, apps provide accessible information and education at minimal costs throughout the cancer care continuum [47]. Apps have various advantages over other traditional approaches, including that they can be referenced even after the consultation and that they require less staff [12,48]. They are accepted by most patients, and they positively contribute to strengthening patients’ engagement and empowerment [44,49-51].

Studies in low- and middle-income countries suggest that mHealth interventions dependent on mobile phone ownership are feasible and may reach the majority of patients in key subgroups, such as those who have low education and limited access to the health system [48,52]. In Argentina, more than 84.2% of women have access to the internet through a smartphone [53]. Reduced access to sexual health counseling has been reported for minority groups [54]; therefore, the implementation of an app-based tool to provide women with information and support might constitute a key intervention for diminishing inequalities in CC prevention.

### Objectives

In this paper, we report results from formative research carried out to understand HPV-tested women’s perceptions about an app as a tool to receive information and support to reduce the emotional impact of HPV-positive results. In addition, we investigated their preferences regarding app design, content, and framing. The analysis was part of a study with the main objective of designing a user-centered counseling app aimed at reducing the psychosocial impact of HPV testing and increasing adherence to follow-up.

## Methods

### Theoretical Foundation

We relied on constructs from the health belief model (HBM) [55] and the integrated behavioral model (IBM) [56]. Both have been successfully used to explain interventions linked to CC prevention and mHealth intervention [57-61]. Following these models, we posited that the intention to adhere to follow-up after a positive HPV test result is determined by changing attitudes (eg, change of fatalistic thoughts about CC prevention and reduced fear), perceived norms (eg, increased value of cancer prevention care), and self-efficacy (eg, reduced perceptions about barriers to CC prevention and increased motivation to perform follow-up procedures) [55,56]. After delivery of HPV test results, the app will provide HPV-positive women with information and emotional and practical support tools that will influence attitudes, perceived norms, and personal agency, thereby favoring functional coping strategies. It will also improve awareness and knowledge of HPV and the importance of continuing diagnosis and treatment, as well as the significance of having the skills to do so (eg, details on how, when, and where). The app can promote cues to action, which will have an effect on individual behavior by reducing the psychosocial impact and triggering an intention to continue health care [55].

**Figure 1:**
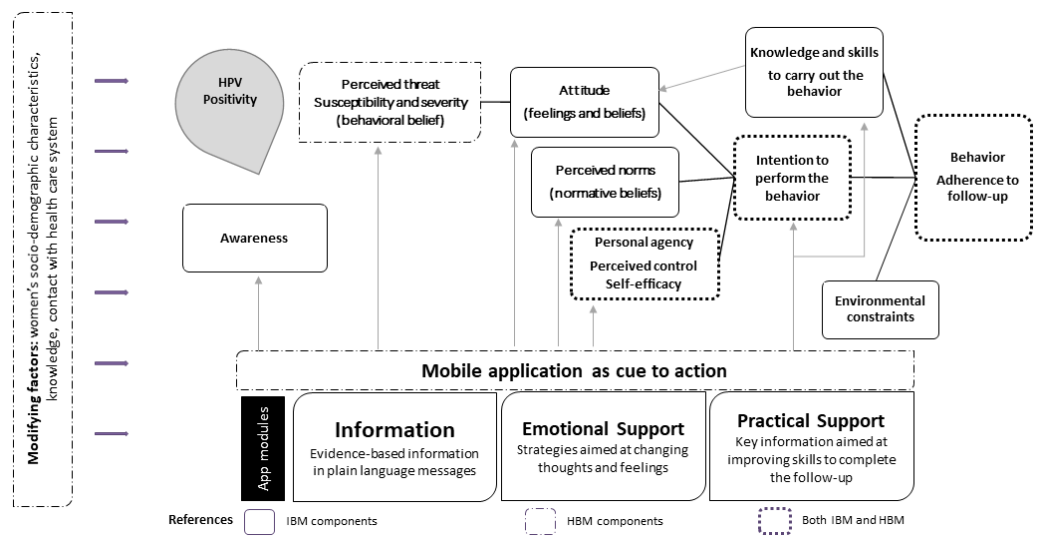
Theoretical foundations of the app’s IA (based on Skinner et al [55] and Montaño et al [56]). HBM: health belief model; HPV: human papillomavirus; IA: information architecture; IBM: integrative behavioral model.

An initial version of the app’s information architecture (IA) based on the theoretical framework consisted of 3 modules (Figure 1): (a) an Information module to provide women with evidence-based information in plain language messages; (b) an Emotional Support module to provide HPV-positive women with support in a way that will allow them to change negative thoughts [7,62] that lead to distress, diminished motivation, and less active self-care; and (c) a Practical Support module to provide women with tools to facilitate the continuation of the line of care, including reminders for diagnostics and treatment consultations.

The app’s design process was based on a user-centered design (UCD) approach [63]. The UCD includes the end users’ participation as co-designers and considers their specific cultural, social, and economic background to understand their preferences of information organization, features, and navigation flow (sequences of screens and IA) [64,65].

### Design of App Mock-ups

The content and labels of the app draft screens (ie, mock-ups) were designed in relation to the HBM and IBM frameworks. We also incorporated findings of a review we carried out to identify mHealth studies reporting on the development or evaluation of mHealth app components [20,59,66].

**Figure 2:**
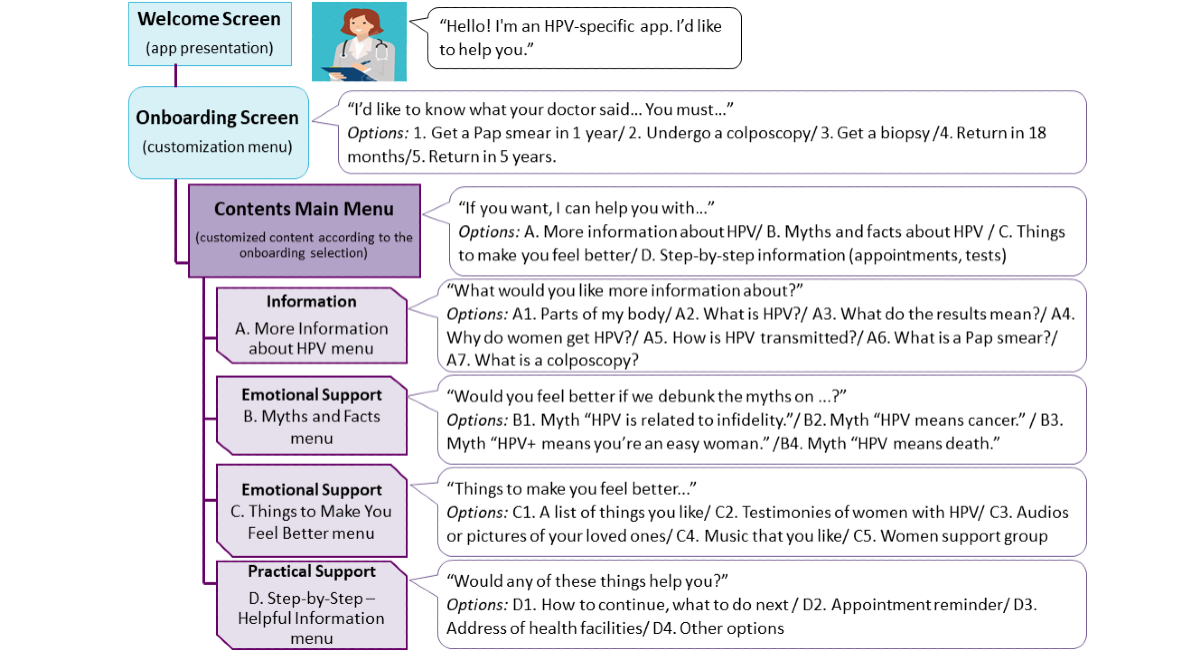
Options for app screens and their sequence and organization. HPV: human papillomavirus; Pap: Papanicolaou.

The draft mock-ups (Figure 2) consisted of 7 screens, including labels of the potential content, menus of options, and draft illustrations, as follows:

1. Welcome screen: presents the app and its purpose.
2. Onboarding screen: inquires about the medical indication received after the HPV-positive result. It offers the following as possible answers: “You must get a Papanicolaou (Pap) smear in 1 year,” “You must undergo a colposcopy,” “You must get a biopsy,” “You must return in 18 months,” and “You must return in 5 years.” The list of responses to this question was based on the guidelines for the prevention of CC established by the health authorities in Argentina [5].
3. Main Menu screen: presents a list of 4 options (Information, Myths and Facts, Things to Make You Feel Better, and Step-by-Step - Helpful Information). The content included on these screens was created from evidence provided by previous studies on women's knowledge and perceptions of HPV, as well as their information needs [6-8,67,68].
4. Information screen: presents a list of topics that the app provides information about.
5. Myths and Facts screen: presents a list of misconceptions about HPV and CC. The app provides information that refutes each myth.
6. Things to Make You Feel Better screen: provides a list of coping strategy-oriented activities.
7. Step-By-Step - Helpful Information screen: We proposed a menu with options to access useful information about the studies and to schedule reminders.

### Setting

The study took place in Ituzaingó, a district located 10 km (6.2 miles) west of the city of Buenos Aires, Argentina. Ituzaingó is part of the Metropolitan Area of Buenos Aires where one-third of the country’s population lives. In 2015, HPV testing was established as a primary screening for women aged 30 years or over who were treated by the public health system. The public health system provides health care access to the population not covered by the social security sector (workers in the informal economy and their families). For the uninsured, health services are free of cost.

### Participants

Eligible women were literate, aged 30 years or over, resided in Ituzaingó, and mobile phone users. We carried out a purposive sampling procedure among women HPV-tested in the past 12 months in the public health care sector. We recruited participants by phone calls during which a recruiter explained the study aims and asked for informed consent. If the woman accepted, she consented to participate in the interview and a focus group (FG). We used the Zoom platform because it allowed women to participate without sharing personal data or logging in. We used age as a stratification criterion because it was considered a crucial variable to understand differences in cellphone use [69]. However, we did not find differences among analyzed groups. The FGs conducted were:

- FG1: 6 women aged 30-49 years
- FG2: 4 women aged ≥50 years
- FG3: 3 women aged 30-49 years
- FG4: 6 women aged ≥50 years.

### Data Collection

The original research protocol with face-to-face FGs was designed before the onset of the COVID-19 pandemic. Due to social distancing measures, we switched to an online strategy. Online tools for data collection are suitable for sensitive topics and allowed us to solve logistical issues [70]. However, according to the literature [70,71], FGs using videoconference software require shorter durations and fewer participants per group. In addition, promoting interactions between participants is more challenging than in face-to-face FGs, due to audio delays or interruptions produced by weak internet connections. Therefore, from the original FG guideline, we selected some dimensions to be collected in an individual interview where women were asked about personal experiences and sensitive topics. We carried out individual online semistructured interviews about (1) the participant’s profile; (2) use of cellphone, apps, and the internet; (3) personal experiences in the gynecological consultation; (4) information needs regarding HPV/CC; (5) risk perception and attitudes of HPV and CC; (6) perceived norms and self-efficacy to adhere to follow-up procedures; and (7) perceptions regarding an app as a tool to receive HPV testing–related information and support. The interviews also helped instruct women in the use of a virtual platform during the FGs, if necessary.

Second, we carried out 4 online FGs using the same virtual platform. Discussion in the FGs began with general questions on women’s knowledge, beliefs, and attitudes regarding HPV results and CC prevention. This information was used as a trigger to introduce questions about women’s preferences regarding the app and contextualize their answers. We then shared the draft app's mock-ups (Figure 2). This allowed us to give the women understandable triggers to debate the concepts presented on each screen. We asked the women about each screen’s content and their preferences for app features. This paper presents the results we obtained from these FGs.

Two trained female researchers collected the information from the FGs in Spanish. One of these women has a background in social sciences and acted as a moderator (author VSA), while the other woman, who has a background in app design and user-experience research (author DS), acted mainly as an observer but added further questions when needed; neither of them lived in Ituzaingó, nor did they have any relation with the health care institutions or their authorities. This was also stated during the FGs. At the end of each FG and if needed, we provided women with accurate information regarding HPV/CC prevention and answered their questions to reduce confusion related to discussed topics.

We carried out the fieldwork during the COVID-19 pandemic (November and December 2020). In that period, social distancing and semilockdown measures were in force for nonessential activities, including most services for nonurgent health issues. Despite this unprecedented context, we recruited 29 women during the individual interviews, of whom 19 (66%) participated in the 4 FGs. We also carried out a pilot FG to test the guide in a virtual environment. Each FG lasted 1 hour 55 minutes on average. Both were digitally recorded to transcribe verbatim.

### Analytic Approach

FG audios were transcribed to carry out thematic analysis of the debates [72], based on an iterative and flexible process following 6 steps:

1. To ensure coding reliability, 2 researchers (authors VSA and LS) become independently familiar with the data through transcriptions and the video recording.
2. We classified data using an initial codebook based on the theoretical constructs (eg, knowledge, beliefs, and attitudes), and in accordance with our research objectives, we identified the reactions to the app and to each draft mock-up screen (opinions).
3. We analyzed each category to generate new themes (eg, “a salient aspect of the data in a patterned way, regardless of whether that theme captures the majority experience of the participants” [72]).
4. Both researchers met to review themes to identify consistencies and resolve the inconsistencies with the other members of the team.
5. We grouped the emergent themes according to their conceptual similarities to define and name the subthemes.
6. We sought examples that adequately graphed each theme. Women’s preferences, reactions and opinions regarding the app draft screens were coded into emergent categories (subthemes) that identified their preferences with regard to content, tool, feature, and design aspect.

For data processing, we used the RQDA package from R software (R Core Team) [73,74] to organize, code, and summarize patterns. RQDA is a tool that assists the analysis of textual data, and it includes a number of standard computer-aided qualitative data analysis features, such as character-level coding, creation of documents or codes memos, and organization of codes into code categories.

To ensure coding reliability, a third author (SA) verified coding against a sample of transcripts and critically reviewed the data and themes to improve the trustworthiness of the study [75]. A detailed description of all procedures used to guarantee the trustworthiness of data collection and analysis is included in Multimedia Appendix 1.

Method details and FG results are presented following the Consolidated Criteria for Reporting Qualitative Research (COREQ).

The study’s protocol was approved by the Diagnóstico por Imagen Morón (DIM) private clinic’s ethics committee. Before the study began, women provided orally informed consent, which was audio-recorded for documentation. The anonymity of participants was guaranteed at each step of the study.

## Results

### Characteristics of the Women

A total of 19 women participated in 4 FGs. Most of them had secondary-level education or less (12/19, 63%) and were employed (11/19, 58%). Most of them lived with a partner and had children, and near three quarters of them had public health insurance (Table 1).

**Table 1:** Characteristics of the focus group participants (N=19).

Variables		n (%)
**Age (years); mean 47.4 years, range 31-66 years**		
	30-39	5 (19)
	40-49	4 (21)
	50+	10 (60)
**Educational level**		
	Secondary (incomplete and complete)	12 (63)
	Tertiary incomplete	5 (26)
	Tertiary complete	2 (11)
**Economic activity status**		
	Economically active (labor force)	14 (74)
	Economically inactive (out of labor force)	5 (26)
**Family status**		
	In a relationship with children	9 (47)
	Single with children	8 (42)
	Single without children	2 (11)
**HPV^a^/Pap^b^** **results**		
	Positive/Normal	13 (68)
	Positive/Abnormal	4 (21)
	Negative	2 (11)
**Health insurance**		
	Public	14 (74)
	Private/social security	5 (26)

^a^HPV: human papillomavirus.

^b^Pap: Papanicolaou.

### Knowledge, Beliefs, and Attitudes Regarding HPV and CC

Most of the participants were unclear on certain aspects related to HPV and CC. HPV transmission was the topic that generated the most questions and caused the most confusion. Although most of the women knew about the sexual transmission of HPV, some mentioned other possible routes of transmission, such as poor hygiene or sharing clothes. The women also asked questions, such as who is at risk of HPV, what type of organism HPV is, what its symptomatology is, what the prognosis is in the case of HPV-positive results, and follow-up or treatment required. They also expressed interest in vaccines and recommended screening tests according to age. The lack of information led 1 of the women to conclude, “We don't know anything” [FG3].

It would be great to have information because sometimes they say, “No, I won't do this to you because of such and such protocol,” and you’re just left thinking, “But how come they did it to my friend?”

FG3, 30-49 years old

Likewise, in the FGs, results identified recurrent beliefs in the different groups, such as that the virus affects more young women or women of childbearing age, that it is transmitted in the context of heterosexual relations, and the idea of dormancy of the virus associated with cancer being something that is awakened.

The concerns that the participants mentioned were related to infidelity, partner conflict in the face of an HPV-positive result, possible discomfort during sexual intercourse due to HPV, or the consequences on fertility. In at least 2 of the groups, the women had initially mistaken the positive HPV test result for a cancer diagnosis.

In all groups, receiving the HPV-positive result triggered negative emotions, such as distress, anger, anxiety, shame, and fear. Those who had a medical consultation where they received information reported that it was a satisfying experience and reduced their concerns. However, those who did not get an opportunity to speak with a doctor shared a lot of concern, which was only increased by the difficulties in obtaining an immediate appointment during the COVID-19 pandemic.

[After receiving the HPV+ result] . . . I spent entire nights crying, days with swollen eyes because I didn't know what I had. And I couldn't rely on a professional, there was nobody open for an appointment, it was terrible because it was just when the pandemic started. They didn't take appointments anywhere and only cared about COVID cases, but people also have other issues.

FG2, 50 years and over

### App Acceptability

Participants in the different FGs expressed their willingness to use an app to access information about HPV and CC, especially to clarify doubts and have access to quality information until they could consult a health professional. In all the groups, the participants indicated that the health authorities, such as the Ministry of Health or the Health Secretariat of the municipality, would be reliable sources that would give legitimacy to the content of the app. The presence of institutional logos would provide an endorsement and, therefore, trust. Most of the participants indicated that they would trust the app if a physician or health professional recommended its use.

Woman 1: The idea [of the app] is great. Yes, I would download it, but if someone recommends it to me...

Moderator: Who has to recommend it?

Woman 1: The doctors.

Woman 2: Well, if the doctor recommends it, even better.

Woman 3: Yes, if it’s the doctor, even better.

Woman 2: Yes, I agree with the ladies. Professionals that come from the Ministry of Health and are approved for everything.

FG1, 30-49 years old

### Preferences on App Content, Features, and Design

#### Communication Style

All groups mentioned that the app should address the user's doubts, offer advice, and provide information in a way that is easy to understand: “It should be like a psychologist; it should listen but also give you advice, guide you” (FG1) and “It should explain things clearly” (FG2).

[How should the app be?]

Woman 1: . . . It should know how to emotionally support us and how to respond to our questions. It should know how to give the user peace of mind and the information they need.

Woman 2: It has to seem human . . .

FG4, 50 years and over

#### The Welcome Screen

This screen included the sentence “Hi, I'm an HPV-specific app. I'd like to help you” as an introduction to the app. Several women criticized that it says, “I’m an app,” and suggested that it should have a person's name, otherwise they would discard its use. Some positively valued the illustration that was included the draft, a health professional taking notes, which was interpreted as a receptive gesture: “Like they’re listening to what you tell them” (FG1).

Woman 1: I would put, instead of “I’m an app,” I would give it a name to generate a more personal bond more human, you know?

Woman 2: Yes, because the greeting “I’m an app” sounds like a robot, and you delete [an app] . . . so they have a human identity, that looks like, simulates being human.

FG3, 30-49 years old

#### The Onboarding Screen

Faced with the options presented on the screen (Figure 2), several women expressed confusion:

Woman 1: I don’t understand . . . so you open the app, and it says it’s going to help you, and now it jumps to “What did the doctor say?”

Woman 2: . . . So, you open the application knowing that you have HPV?

Woman 1: . . . I think there could be another [screen] before asking, “Did you get an HPV test?”

FG2, 50 years and over

Some women were unaware of the different tests listed: “What would be the difference between a Pap smear and colposcopy?” (FG3). Regarding their results, several women had been told that “everything was fine, that I should come back in a year” (FG1), even though they registered an HPV-positive result. This fact influenced the women’s reaction as to which option they should choose, causing doubts as to whether the doctor's indication was the right one for their case.

Woman 1: I was told that I have to get a PAP [smear] and colpo[scopy] every year, as well as a mammogram, which doesn’t appear here [as an option on the screen].

Woman 2: [Reacting with doubts regarding the screen] . . . Sometimes you think you have to undergo such and such study, and you don’t actually have to undergo it, and you never know if that’s right or not.

FG3, 30-49 years old

#### The Main Menu Screen

The women's reaction to the Main Menu screen (Figure 2) was positive, with a high level of acceptability of the options: ”I would like to read everything; if I open [the app], in just a day I’d explore the whole application, from top to bottom“ (FG3).

Woman 1: It’s great.

Woman 2: I think it’s good.

Woman 3: I think it’s very good.

Woman 4: I would separate ”If you like, I can help you with . . .“ and you have all the information, ”Would you like more information?“ and then, absolutely, refer you with an appointment . . . make an appointment to have the study done, no matter what, and to consult a specialist, always.

FG4, 50 years and over

#### The Information Screen

When evaluating the Information screen (Figure 2), the women agreed that all the contents displayed were particularly important and interesting to them. Moreover, these contents led to proposals from the women on topics that this module of the app should address.

[The app should provide information about] ”call or contact us if you have any symptoms,“ or give guidelines on at what age you should do it . . . ”If you are over 30 years old, you have to get a specialized test.“ In other words, more controls, to have all that at your fingertips. ”Babies must be vaccinated from this age to that age“ . . . That would help us a lot.

FG2, 50 years and over

#### The Myths and Facts Screen

This screen generated both positive and negative reactions. Among the positive comments, the women recognized that debunking inaccurate beliefs reduces concern: ”It’s important to debunk myths because they generate a lot of stress“ (FG4). Among those who rejected the Myths and Facts screen, it was observed that the contents were interpreted as statements about their behaviors, not as a myth to be refuted. In some cases, the contents generated discomfort: ”It's like they blame us“ (FG1).

[After reading the screen] . . . You don’t have to be unfaithful or suffer from . . . it isn’t cancer . . . nor is it because the person is an easy woman . . . nor did they die; in other words, they live with HPV and nothing else . . . I mean, I tested positive, but I wasn’t unfaithful or anything like that; I got it and I don’t know where. It could have been in a gynecological study that I had done . . .

FG2, 50 years and over

#### The Things to Make You Feel Better Screen

This screen proposed a list of activities so that the user could find emotional support. Among the options on the screen, women were asked to indicate which options they preferred (Figure 2). Of the proposed alternatives, the favored ones were ”Listening to other women's stories“ and finding ”Women support groups that help each other.“ Some participants stated that they wanted to hear testimonies from other women to know ”how she coped with the HPV result“ and ”how it went“ (FG3). They also suggested that the app provide statistics about women with HPV ”who got cured because they did everything“ (FG3).

The other proposed activities, such as ”List of activities you like,“ ”Listen to messages or see pictures of loved ones,“ and ”Have music you like,“ were discarded because, on the one hand, “People already have [music or photos] on their cell phone” (FG1), while, on the other hand, they generated doubts about the personal information shared with the app.

Woman 1: [The “Things to Make You Feel Better” screen] . . . I don't get it. In the app, are you going to hear or see stories of other women who went through the same thing? That's good. But . . . “Listen to messages from your loved ones” what would there be there? Audios in there, or is that something you would have to add? Have music that you like to listen to? I kind of don't relate it to the issue . . .

Woman 2: I already know what makes me feel better; why remind me of it?

Woman 3: Would we put pictures on the app? I wouldn't let it, for example . . . [when] it asks for permissions to access [meaning not giving permission to access photos or files].

FG4, 50 years and over

#### The Step by Step - Helpful Information Screen

The women of all the FGs named the Reminders function as the most relevant: “I think it's great that it notifies you that you have to have a check-up” (FG4). Likewise, many participants proposed including a directory with care centers specialized in CC prevention. They also proposed a function that would allow scheduling appointments or “to be able to make an appointment directly there [in the app], you can enter your address, and they will give you an appointment at the nearest clinic” (FG1).

Several women showed interest in receiving notifications of the availability of results: “so that you can go and know that it’s ready” (FG2). Others proposed including a function that would allow them to store or record previously performed studies: “. . . maybe the doctor asks me and I don't remember (. . .) I have to go and rummage through all the papers to see when [the last study] was” (FG2).

. . . [in the app] you list the tests you should get done, you have the doctors' appointments, and you go . . . you have an agenda where you have all the tests you should get, so, well, you check off: “I’ve already had this done, ah, I need . . .” I don't know, “next time I go to the doctor's office, I’ll ask them if this test is suitable or not,” but you already have an agenda to follow and comply with.

FG3, 30-49 years old

#### Screen Flow

One criticized aspect of the draft shown to the women was the sequence in which the screens were displayed.

Woman 1: I would like that before choosing an option on “What did the doctor say?,” it would say, “Do you know what HPV is?” first. Then if they don't know, send the user to an option where you give her all the information. If she already knows what it is, then follow with the “What did the doctor say?” options.

Woman 2: I want to find out what [HPV] is all about first, then I want to see “What method should I follow?” Or, if I already know what I should do, ”What is [that test] for?“

Woman 1: Also . . . if you’re already in treatment, ”What step are you in?,“ the ”step by step“ of the process, the appointments and the studies would be missing. If you’re already in treatment, ”How are you?“ Give other options there: ”How are you feeling?,“ ”Are you going to a center?,“ ”Are you talking to anyone?“ That would also be another bonus point, after the step by step.

FG2, 50 years and over

#### Format Preferences

Regarding the different contents proposed in the app, the women proposed videos with explanations provided by professionals and testimonial videos with women relating their cases and evolution. They pointed out the need for a feature that would also allow sharing the information in the app with other women.

As for formats, women positively valued infographics and illustrations and, to a lesser extent, texts. Two groups stressed the importance of providing content using different formats to ensure accessibility: texts with audio for people with visual impairment or videos with text for those with hearing impairment.

Regarding the use of images, opinions were divided. On the one hand, there were some who argued that health contents through realistic images ”are shocking“ (FG1) and ”are very off-putting“ (FG4). On the other hand, women who were in favor of using real images pointed out that ”if they’re real, they help to raise awareness“ (FG2).

Woman 1: I would like there to be a video with a specialist who can clearly explain what this disease is and what steps you have to follow or what has to be done . . . so that a registered specialist can guide you. Not just anyone talking . . . and that they go straight to the point . . . In the general framework, they can guide you and tell you what you can do.

Woman 2: . . . but women's opinions too, there are some videos . . . of real cases, it would be good too, real cases . . .

FG3, 30-49 years old

Table 2 summarizes the themes and subthemes obtained during analysis. Additionally, in Multimedia Appendix 1, we present verbatim examples for each theme and subtheme.

**Table 2:** Knowledge, attitudes, and beliefs, acceptability, and screen evaluation: the app’s content and features (themes and subthemes).

Themes	Subthemes	Subtheme definition
**Knowledge**		
	Lack of information	The questions and concerns the women expressed during FGs^a^ that showed a lack of general knowledge of HPV^b^ and CC^c^ prevention
	Partial or incorrect information about HPV transmission	The questions and statements the women expressed during FGs that showed incomplete or incorrect information about HPV transmission
	Information needs: nature of HPV	The questions and concerns the women expressed during FGs regarding the nature of HPV (“What kind of disease is it?”)
	Information needs: symptomatology	The questions and concerns the women expressed during FGs regarding possible HPV symptoms
	Information needs: prognosis	The questions and concerns the women expressed during FGs regarding the forecasting of the probable course of HPV and the chances of recovery
	Information needs: who is at risk for HPV?	The questions and concerns the women expressed during FGs regarding who is at risk for HPV (what kind of person or age/gender/sexual behavior profile)
	Information needs: follow-up medical procedures	The questions and concerns the women expressed during FGs regarding the follow-up medical procedures required after an HPV-positive result
**HPV beliefs**		
	Only affects young people	Narratives showing women’s perceptions on HPV as a disease that only affects young people
	Is only transmitted via heterosexual intercourse	Narratives showing women’s perceptions on HPV as a disease that is only transmitted in heterosexual intercourse
	HPV and cancer as dormant diseases	Narratives showing women’s perceptions on the asymptomatic nature of HPV linked with the idea of cancer as a dormant disease
**Attitudes**		
	An HPV-positive result causes conflicts with partner over infidelity.	Emotional reactions related to the HPV-positive results (such as anger) that may cause conflict with partners due to suspicions of infidelity
	An HPV-positive result causes fear of consequences in sexual intercourse.	Narratives around fear of having consequences in sexual intercourse due to HPV-positive results (such as feel pain or discomfort)
	An HPV-positive result causes concerns on infertility.	Narratives around concerns that HPV causes infertility
	An HPV-positive result causes distress.	Narratives around concerns due to distress caused by an HPV-positive result
**App acceptability**	Trust	Reactions to the app in terms of acceptability and the criteria to be considered trustworthy
**Welcome screen**	App preferences identity: age/gender	Preferences around the app’s character identity in terms of age and gender
**Onboarding screen**	Reactions: confusion	Women’s comments, concerns, and questions that showed the confusion produced by the Onboarding screen
**Contents Menu screen**	Reactions: positively evaluated content	Reaction expressed in terms of approving the displayed content
**Information screen**	Reactions: positively evaluated content	Reaction expressed in terms of approving the displayed content
**Myths and Facts screen (Emotional Support module)**		
	Accepts the Myths and Facts screen	Reaction expressed in terms of approving the displayed content
	Rejects the Myths and Facts screen	Reaction expressed in terms of rejecting the displayed content (includes other reactions such as confusion or misunderstanding of the displayed content)
**Things to Make You Feel Better screen (Emotional Support module)**		
	Preferred activity: listening to other women	The main activities the women preferred from a list of displayed options
	Preferred activity: sharing with other women	The main activities the women preferred from a list of displayed options
	Dismissed activities	The activities dismissed as app features
**Step by Step - Helpful Information screen (Practical Support module)**		
	Reminder to make an appointment	App features proposed/accepted as practical support
	Results availability reminder	App features proposed/accepted as practical support
	Reminder of having an appointment	App features proposed/accepted as practical support
	Directory	App features proposed/accepted as practical support
**General app design**		
	Communication style	Preferences on the app’s contents communication style (how it should be)
	Contents organization: screen flow	Preferences and suggestions on the app’s screens sequence (order in which screens must be displayed in the app according to its content)
	Formats preferences: videos	Preferences on the app’s content formats
	Formats preferences: multiple formats for accessibility	Preferences on the app’s content formats
	Formats preferences: images/photographs	Preferences on the app’s content formats

^a^FG: focus group.

^b^HPV: human papillomavirus.

^c^CC: cervical cancer.

## Discussion

### Principal Results

Our results showed that women positively valued the app as a tool to obtain information and counseling about HPV and CC. Similar findings were reported by studies that analyzed women’s preference for apps aimed at providing support to women diagnosed with cancer [43,76-80]. Two studies analyzed the acceptability of an app oriented toward educating people on HPV and CC prevention. A research team from Norway designed an app called FightHPV [81]. The app is a digital game-based learning tool for mobile devices that aims to communicate concepts that help people understand HPV-related diseases, such as CC, and their prevention. Authors reported positive reactions from potential users toward the educational game (they enjoyed the game, and the game was challenging). Additionally, a study carried out in the United States reported the development of an interactive virtual patient educator for Hispanic women about CC and HPV. During the design process and in the pilot study, participants reported high levels of satisfaction with the interaction with the system [82].

Our app was conceived to provide support to HPV-tested women after the delivery of the test results. Our study showed that women would accept and trust its contents as long as a physician recommended it. Greenhalgh et al [83] analyzed dimensions that influence the diffusion and adoption of health innovative interventions. They conducted a systematic review, and many of their conclusions were used to develop the Consolidated Framework Intervention Research (CFIR). Following Greenhalgh et al’s [83] conclusions, the CFIR states that a key element to achieving the acceptability and adoption of health innovations is leader endorsement during its implementation. According to this finding, experts' opinions have a particular influence on the beliefs and actions of those who have to adopt the health innovation. In our case, health professionals can be perceived as experts who may exert influence through their authority and status, instilling credibility to the contents provided by the app (legitimacy).

Evidence has shown that it is important to offer users personalized contents [84]. Initially, we chose an onboarding screen to provide customized content according to the clinical screening, diagnosis, and treatment protocol recommended by the National Ministry of Health and the National Cancer Institute [5]. However, our study concluded that women find the onboarding options based on these recommendations confusing, as they cannot relate them to the explanation provided by the health professional. Adherence of health professionals to national screening-diagnosis-treatment recommendations is rather low [3], so women are often given instructions on follow-up that do not align with national guidelines. This is the case, for example, of the woman who was told to repeat a Pap smear and colposcopy annually, an option not endorsed by the national guidelines. In addition, evidence shows that women lack general knowledge on HPV and CC prevention and have problems understanding what their health professionals tell them [39]. Therefore, our study showed the importance of an initial screen that provides a menu to access information regarding HPV, and the purpose of screening tests before providing customized content.

Following our theoretical framework, the app initial draft included 3 separate modules: Information, Emotional Support, and Practical Support. However, the FG participants rejected this scheme and suggested reorganizing the screens and a new hierarchical organization of modules. They agreed that more knowledge helps them face fears and that the emotional support tools (eg, Things to Make You Feel Better) should complement the provided information, not be the central content of the app. This result enhances the importance of user perceptions of the app content and its organization as a key element in the design process in order to ensure high final user engagement [78].

Our study showed that women highly value having a medical appointment reminder feature (Practical Support module). Similarly, the study Application of Communication and Information Technologies to Self-Collection (ATICA study, for its initials in Spanish), conducted in Jujuy, Argentina, found that HPV-positive women highly accept receiving reminders through the Short Messaging Service (SMS) to increase their adherence to a Pap-based triage [85]. This is an important result for the app design as reminders (eg, through SMS) have been found to improve medication adherence and other treatment compliance [48].

Regarding the app style and tone, women asked for an empathic communication style even when clearly acknowledging that they were interacting with a device (mobile phone). Similar findings were found in a previous study that analyzed women’s preferences regarding SMS content design [86] to receive information about triage after an HPV-positive result. In that study, also carried out in Argentina, women requested the SMS content to emulate the overall friendly style of community health workers. Other authors found that empathy is positively valued among women when receiving information about being HPV-positive and follow-up [39]. Additionally, our results found that women positively value the application as it can provide information through multiformat content. They agreed that subtitled videos, illustrations, or infographics with audio help them understand the content while ensuring accessibility. However, we did not find agreement on preference related to real pictures or only text content.

Our findings have some implications for the design of mHealth interventions targeted toward HPV-tested women. Health care providers may consider using electronic health (eHealth) technologies, such as smartphone apps, to provide patients with psychosocial support after HPV-positive result delivery. Furthermore, policy makers may consider funding and supporting evidence‐based interventions delivered through eHealth platforms. This is particularly important in the midst of the COVID-19 pandemic, as using mHealth strategies has been signaled as a key intervention that may help women in a context where we must minimize face-to-face encounters.

### Limitations

This study had some limitations. Due to the COVID-19 pandemic, we conducted online FGs, and we reduced the number of participants for each group. The small sample and the specific study setting may have limited the generalizability of our findings. However, it is considered sufficient for qualitative research, and clear themes emerged from the data. However, the pandemic created an optimal setting as it hinders physical interaction, thereby strengthening the argument for an mHealth approach. In addition, we achieved theoretical saturation to the main findings, such as content presence and screen organization. The study results were limited to the women's points of view and did not include health providers’ opinions regarding the app; however, we plan to interview physicians from the Ituzaingó health system as part of the app process design.

### Conclusion

This formative research has shown that women accept an app that provides information and counseling as part of the health provider-woman encounter. A lack of knowledge or misinformation about HPV and CC was the core of their opinions and demands regarding the app design. Our findings highlight the need to involve end users in the early stages of the conceptualization and design process of mHealth innovations. The key elements of the app design must be carefully chosen in an endeavor to guarantee both comprehension of the contents and usefulness to the end user.

## Appendix

Multimedia Appendix 1 Thematic analysis: results and procedures.

## Abbreviations

CC: cervical cancer
CFIR: Consolidated Framework Intervention Research
eHealth: electronic health
FG: focus group
HBM: health belief model
HPV: human papillomavirus
IA: information architecture
IBM: integrative behavioral model
mHealth: mobile health
Pap: Papanicolaou
SMS: Short Messaging Service
STI: sexually transmitted infection
UCD: user-centered design

## References

[1] Bray F, Ferlay J, Soerjomataram I, Siegel RL, Torre LA, Jemal A (2018). Global cancer statistics 2018: GLOBOCAN estimates of incidence and mortality worldwide for 36 cancers in 185 countries. CA Cancer J Clin.

[2] Murillo R, Almonte M, Pereira A, Ferrer E, Gamboa OA, Jerónimo J, Lazcano-Ponce E (2008). Cervical cancer screening programs in Latin America and the Caribbean. Vaccine.

[3] Arrossi S, Paolino M, Sankaranarayanan R (2010). Challenges faced by cervical cancer prevention programs in developing countries: a situational analysis of program organization in Argentina. Rev Panam Salud Publica.

[4] Lazcano-Ponce E, Lorincz AT, Cruz-Valdez A, Salmerón J, Uribe P, Velasco-Mondragón E, Nevarez PH, Acosta RD, Hernández-Avila M (2011). Self-collection of vaginal specimens for human papillomavirus testing in cervical cancer prevention (MARCH): a community-based randomised controlled trial. Lancet.

[5] Arrossi S, Thouyaret L, Paul L (2020). Prevención Del Cáncer Cervicouterino: Recomendaciones Para El Tamizaje, Segui Miento y Tratamiento de Mujeres En El Marco de Programas de Tamizaje Basados En El Test de VPH.

[6] Rask M, Swahnberg K, Lindell G, Oscarsson M (2017). Women's experiences of abnormal Pap smear results: a qualitative study. Sex Reprod Healthc.

[7] Arrossi S, Almonte M, Herrero R, Gago J, Sánchez Antelo V, Szwarc L, Thouyaret L, Paolino M, Wiesner C (2020). Psycho-social impact of positive human papillomavirus testing in Jujuy, Argentina results from the Psycho-Estampa study. Prev Med Rep.

[8] Verhoeven V, Baay MF, Baay PE, Lardon F, Van Royen P, Vermorken JB (2010). Everything you always wanted to know about HPV (but could not ask your doctor). Patient Educ Couns.

[9] Marván Ma Luisa, Ehrenzweig Y, Catillo-López Rosa Lilia (2016). Fatalistic beliefs and cervical cancer screening among Mexican women. Health Care Women Int.

[10] Barnack-Tavlaris JL, Serpico JR, Ahluwalia M, Ports KA (2016). "I have human papillomavirus": an analysis of illness narratives from the Experience Project. Appl Nurs Res.

[11] Lehmann U, Sanders D (2007). Community Health Workers: What Do We Know About Them? The State of the Evidence on Programmes, Activities, Costs an Impact on Health Outcomes of Using Community Health Workers.

[12] Kannisto KA, Koivunen MH, Välimäki MA (2014). Use of mobile phone text message reminders in health care services: a narrative literature review. J Med Internet Res.

[13] Arrossi S, Thouyaret L, Herrero R, Campanera A, Magdaleno A, Cuberli M, Barletta P, Laudi R, Orellana L (2015). Effect of self-collection of HPV DNA offered by community health workers at home visits on uptake of screening for cervical cancer (the EMA study): a population-based cluster-randomised trial. Lancet Global Health.

[14] Binka C, Doku DT, Awusabo-Asare K (2017). Experiences of cervical cancer patients in rural Ghana: an exploratory study. PLoS One.

[15] Karasz A, McKee MD, Roybal K (2003). Women's experiences of abnormal cervical cytology: illness representations, care processes, and outcomes. Ann Fam Med.

[16] Szwarc L, Sánchez Antelo V, Paolino M, Arrossi S (2021). "I felt myself getting sick:" women's perceptions and understandings of a positive human papillomavirus test in Jujuy, Argentina. Salud Colect.

[17] León-Maldonado L, Wentzell E, Brown B, Allen-Leigh B, Torres-Ibarra L, Salmerón J, Billings DL, Thrasher JF, Lazcano-Ponce E (2016). Perceptions and experiences of human papillomavirus (hpv) infection and testing among low-income Mexican women. PLoS One.

[18] Castro VM, Arellano GM (2014). Social support networks and gender: experience of women with HPV, dysplasia and cervical cancer (Spanish). La ventana. Revista de estudios de género.

[19] Monsalve-Páez S, Valderrama-Vega D, Castillo-Zamora MF, Guzmán-Sabogal YR, Amaya-Guío J (2015). Experiencia de las pacientes frente a citología cérvico-vaginal reportada como ASCUS o LEI de bajo grado en dos instituciones de Bogotá (Colombia), 2014. Rev Colomb Obstet Ginecol.

[20] Szwarc L, Sánchez Antelo V, Paolino M, Arrossi S (2021). "I'm neither here, which would be bad, nor there, which would be good": the information needs of HPV+ women. A qualitative study based on in-depth interviews and counselling sessions in Jujuy, Argentina. Sex Reprod Health Matters.

[21] Cendejas BR, Smith-McCune KK, Khan MJ (2015). Does treatment for cervical and vulvar dysplasia impact women's sexual health?. Am J Obstet Gynecol.

[22] Leite V, Santos BD, Pereira MG (2019). Psychosocial impact of human papillomavirus on women's sexual dissatisfaction and quality of life. J Psychosom Obstet Gynaecol.

[23] Wang K, Jeng C, Yang Y, Chen C, Cheng W, Chen T, Mast TC, Wang Y, Hsieh C (2010). The psychological impact of illness among women experiencing human papillomavirus-related illness or screening interventions. J Psychosom Obstet Gynaecol.

[24] Kahn JA, Slap GB, Bernstein DI, Tissot AM, Kollar LM, Hillard PA, Rosenthal SL (2007). Personal meaning of human papillomavirus and Pap test results in adolescent and young adult women. Health Psychol.

[25] Waller J, Marlow LAV, Wardle J (2007). The association between knowledge of HPV and feelings of stigma, shame and anxiety. Sex Transm Infect.

[26] D'Egidio V, Sestili C, Mancino M, Sciarra I, Cocchiara R, Backhaus I, Mannocci A, De Luca A, Frusone F, Monti M, La Torre G, RETURN TO BREAST Collaborative group (2017). Counseling interventions delivered in women with breast cancer to improve health-related quality of life: a systematic review. Qual Life Res.

[27] Lancaster T, Stead LF (2017). Individual behavioural counselling for smoking cessation. Cochrane Database Syst Rev.

[28] Broutet N, O’Neal Eckert L, Ullrich A, Bloem P (2014). Comprehensive Cervical Cancer Control: A Guide to Essential Practice, 2nd Ed.

[29] Wang Z, Lau JTF, Ip M, Ho SPY, Mo PKH, Latkin C, Ma YL, Kim Y (2018). A randomized controlled trial evaluating efficacy of promoting a home-based HIV self-testing with online counseling on increasing HIV testing among men who have sex with men. AIDS Behav.

[30] Coates TJ, Richter L, Caceres C (2008). Behavioural strategies to reduce HIV transmission: how to make them work better. Lancet.

[31] Kurth AE, Chhun N, Cleland CM, Crespo-Fierro M, Parés-Avila JA, Lizcano JA, Norman RG, Shedlin MG, Johnston BE, Sharp VL (2016). Linguistic and cultural adaptation of a computer-based counseling program (CARE+ Spanish) to support HIV treatment adherence and risk reduction for people living with HIV/AIDS: a randomized controlled trial. J Med Internet Res.

[32] Pan American Health Organization (2016). Integrating HPV Testing in Cervical Cancer Screening Program: A Manual for Program Managers.

[33] Santarossa S, Kane D, Senn CY, Woodruff SJ (2018). Exploring the role of in-person components for online health behavior change interventions: can a digital person-to-person component suffice?. J Med Internet Res.

[34] Evans C, Nalubega S, McLuskey J, Darlington N, Croston M, Bath-Hextall F (2016). The views and experiences of nurses and midwives in the provision and management of provider-initiated HIV testing and counseling: a systematic review of qualitative evidence. JBI Database Syst Rev Implement Rep.

[35] Rodríguez Torres A, Jarillo Soto EC, Casas Patiño D (2018). Medical consultation, time and duration. Medwave.

[36] Castro-Vásquez Ma del Carmen, Arellano-Gálvez Ma del Carmen (2010). Access to information by women with HPV, cervical dysplasia and cancer in situ. Salud Publica Mex.

[37] Wiesner C, Acosta J, Díaz Del Castillo A, Tovar S (2012). Social representations of human papillomavirus in Bogotá, Colombia. Med Anthropol.

[38] McRae J, Martin C, O'Leary J, Sharp L, Irish Cervical Screening Research Consortium (CERVIVA) (2014). "If you can't treat HPV, why test for it?" Women's attitudes to the changing face of cervical cancer prevention: a focus group study. BMC Womens Health.

[39] O'Connor M, Costello L, Murphy J, Prendiville W, Martin CM, O'Leary JJ, Sharp L, Irish Screening Research Consortium (CERVIVA) (2015). Influences on human papillomavirus (HPV)-related information needs among women having HPV tests for follow-up of abnormal cervical cytology. J Fam Plann Reprod Health Care.

[40] Paolino M, Campanera A, Martiarena SN, Echenique AL, López N, Gago J, Straw C, Ponce M, Arrossi S (2019). Triage of women with human papillomavirus self-collection in Jujuy Province. Rev argent salud pública.

[41] Fjell M, Langius-Eklöf Ann, Nilsson M, Wengström Y, Sundberg K (2020). Reduced symptom burden with the support of an interactive app during neoadjuvant chemotherapy for breast cancer: a randomized controlled trial. Breast.

[42] Greer JA, Jacobs J, Pensak N, MacDonald JJ, Fuh C, Perez GK, Ward A, Tallen C, Muzikansky A, Traeger L, Penedo FJ, El-Jawahri A, Safren SA, Pirl WF, Temel JS (2019). Randomized trial of a tailored cognitive-behavioral therapy mobile application for anxiety in patients with incurable cancer. Oncologist.

[43] Børøsund E, Ehlers SL, Varsi C, Clark MM, Andrykowski MA, Cvancarova M, Solberg Nes L (2020). Results from a randomized controlled trial testing StressProffen; an application-based stress-management intervention for cancer survivors. Cancer Med.

[44] Hantsoo L, Criniti S, Khan A, Moseley M, Kincler N, Faherty LJ, Epperson CN, Bennett IM (2018). A mobile application for monitoring and management of depressed mood in a vulnerable pregnant population. Psychiatr Serv.

[45] Firth J, Torous J, Nicholas J, Carney R, Rosenbaum S, Sarris J (2017). Can smartphone mental health interventions reduce symptoms of anxiety? A meta-analysis of randomized controlled trials. J Affect Disord.

[46] Firth J, Torous J, Nicholas J, Carney R, Pratap A, Rosenbaum S, Sarris J (2017). The efficacy of smartphone-based mental health interventions for depressive symptoms: a meta-analysis of randomized controlled trials. World Psychiatry.

[47] Jongerius C, Russo S, Mazzocco K, Pravettoni G (2019). Research-tested mobile apps for breast cancer care: systematic review. JMIR Mhealth Uhealth.

[48] Hall CS, Fottrell E, Wilkinson S, Byass P (2014). Assessing the impact of mHealth interventions in low- and middle-income countries: what has been shown to work?. Glob Health Action.

[49] Vo V, Auroy L, Sarradon-Eck A (2019). Patients' perceptions of mHealth apps: meta-ethnographic review of qualitative studies. JMIR Mhealth Uhealth.

[50] Shake MC, Crandall KJ, Mathews RP, Falls DG, Dispennette AK (2018). Efficacy of Bingocize: a game-centered mobile application to improve physical and cognitive performance in older adults. Games Health J.

[51] Rosen KD, Paniagua SM, Kazanis W, Jones S, Potter JS (2018). Quality of life among women diagnosed with breast cancer: a randomized waitlist controlled trial of commercially available mobile app-delivered mindfulness training. Psychooncology.

[52] Beratarrechea A, Lee AG, Willner JM, Jahangir E, Ciapponi A, Rubinstein A (2014). The impact of mobile health interventions on chronic disease outcomes in developing countries: a systematic review. Telemed J E Health.

[53] INDEC (2020). Access and use of information and communication technologies (Spanish). EPH. Inf Técnicos Cienc y Tecnol.

[54] Aitken M, Clancy B, Nass D (2017). The Growing Value of Digital Health: Evidence and Impact on Human Health and the Healthcare System (Institute Report).

[55] Skinner C, Tiro J, Champion V, GlanZ K, Rimer BK, Viswanath VK (2015). The health belief model. Health Behavior and Health Education. 5th ed.

[56] Montaño D, Kasprzyk D, Glanz K, Rimer BK, Viswanath VK (2015). Theory of reasoned action, theory of planned behavior, the integrated behavioral model. Health Behavior and Health Education. 5th ed.

[57] Moore de Peralta A, Holaday B, Hadoto IM (2017). Cues to cervical cancer screening among U.S. Hispanic women. Hisp Health Care Int.

[58] Shida J, Kuwana K, Takahashi K (2018). Behavioral intention to prevent cervical cancer and related factors among female high school students in Japan. Jpn J Nurs Sci.

[59] Sánchez AV, Kohler RE, Szwarc L, Paolino M, Viswanath K, Arrossi S (2020). Knowledge and perceptions regarding triage among human papillomavirus-tested women: a qualitative study of perspectives of low-income women in Argentina. Womens Health (Lond).

[60] Fleming K, Simmons VN, Christy SM, Sutton SK, Romo M, Luque JS, Wells KJ, Gwede CK, Meade CD (2018). Educating Hispanic women about cervical cancer prevention: feasibility of a Promotora-LED Charla intervention in a farmworker community. Ethn Dis.

[61] Darville G, Burns J, Chavanduka T, Anderson-Lewis C (2021). Utilizing theories and evaluation in digital gaming interventions to increase human papillomavirus vaccination among young males: qualitative study. JMIR Serious Games.

[62] Moore de Peralta A, Holaday B, McDonell JR (2015). Factors affecting Hispanic women's participation in screening for cervical cancer. J Immigr Minor Health.

[63] Lyon AR, Koerner K (2016). User-centered design for psychosocial intervention development and implementation. Clin Psychol (NY).

[64] Schnall R, Rojas M, Bakken S, Brown W, Carballo-Dieguez A, Carry M, Gelaude D, Mosley JP, Travers J (2016). A user-centered model for designing consumer mobile health (mHealth) applications (apps). J Biomed Inform.

[65] Garrett JJ (2011). The elements of user experience: user-centered design for the web and beyond. Choice Rev Online.

[66] Paolino M, Arrossi S (2011). Women's knowledge about cervical cancer, Pap smear and human papillomavirus and its relation to screening in Argentina. Women Health.

[67] Bush NE, Smolenski DJ, Denneson LM, Williams HB, Thomas EK, Dobscha SK (2017). A virtual hope box: randomized controlled trial of a smartphone app for emotional regulation and coping with distress. Psychiatr Serv.

[68] Christoforou M, Sáez Fonseca JA, Tsakanikos E (2017). Two novel cognitive behavioral therapy-based mobile apps for agoraphobia: randomized controlled trial. J Med Internet Res.

[69] Forgays DK, Hyman I, Schreiber J (2014). Texting everywhere for everything: gender and age differences in cell phone etiquette and use. Comput Hum Behav.

[70] Davies L, LeClair KL, Bagley P, Blunt H, Hinton L, Ryan S, Ziebland S (2020). Face-to-face compared with online collected accounts of health and illness experiences: a scoping review. Qual Health Res.

[71] Flynn R, Albrecht L, Scott SD (2018). Two approaches to focus group data collection for qualitative health research. Int J Qual Methods.

[72] Braun V, Clarke V (2006). Using thematic analysis in psychology. Qual Res Psychol.

[73] Huang Ronggui (2016). What Is RQDA and What Are Its Features?.

[74] R Core Team (2020). R: A Language and Environment for Statistical Computing.

[75] Lincoln YS, Guba EG (1985). Naturalistic Inquiry.

[76] Yanez B, Oswald LB, Baik SH, Buitrago D, Iacobelli F, Perez-Tamayo A, Guitelman J, Penedo FJ, Buscemi J (2020). Brief culturally informed smartphone interventions decrease breast cancer symptom burden among Latina breast cancer survivors. Psychooncology.

[77] Visser A, Prins JB, Jansen L, Radema SA, Schlooz MS, van Dalen T, van Laarhoven HW (2018). Group medical consultations (GMCs) and tablet-based online support group sessions in the follow-up of breast cancer: a multicenter randomized controlled trial. Breast.

[78] Buscemi J, Buitrago D, Iacobelli F, Penedo F, Maciel C, Guitleman J, Balakrishnan A, Corden M, Adler RF, Bouchard LC, Perez-Tamayo A, Yanez BR (2019). Feasibility of a smartphone-based pilot intervention for Hispanic breast cancer survivors: a brief report. Transl Behav Med.

[79] Lubberding S, van Uden-Kraan CF, Te Velde EA, Cuijpers P, Leemans CR, Verdonck-de Leeuw IM (2015). Improving access to supportive cancer care through an eHealth application: a qualitative needs assessment among cancer survivors. J Clin Nurs.

[80] Ormel I, Onu C, Magalhaes M, Tang T, Hughes J, Law S (2021). Using a mobile app-based video recommender system of patient narratives to prepare women for breast cancer surgery: development and usability study informed by qualitative data. JMIR Form Res.

[81] Ruiz-López T, Sen S, Jakobsen E, Tropé A, Castle PE, Hansen BT, Nygård M (2019). FightHPV: design and evaluation of a mobile game to raise awareness about human papillomavirus and nudge people to take action against cervical cancer. JMIR Serious Games.

[82] Mendu S, Boukhechba M, Gordon J, Datta D, Molina E, Arroyo G, Proctor SK, Wells KJ, Barnes LE (2018). Design of a culturally-informed virtual human for educating Hispanic women about cervical cancer. Int Conf Pervasive Comput Technol Healthc.

[83] Greenhalgh T, Robert G, Macfarlane F, Bate P, Kyriakidou O (2004). Diffusion of innovations in service organizations: systematic review and recommendations. Milbank Q.

[84] Bartholomew K, Parcel G, Kok G, Gottlieb N, Fernández M (2011). Planning Health Promotion Programs: An Intervention Mapping Approach, 3rd ed.

[85] Arrossi S, Viswanath KV, Paolino M, Luzcubir P, Martiarena N, Ugarte M, Campanera A, Zalacain Colombo J, Thouyaret L, Cuberli M, Kohler RE, Orellana L (2020). Effectiveness of a multi-component mhealth intervention for triage after HPV self-collection: preliminary results of the ATICA cluster randomized trial.

[86] Sanchez Antelo V, Kohler RE, Curotto M, Viswanath KV, Paolino M, Arrossi S (2020). Developing SMS content to promote Papanicolaou triage among women who performed HPV self-collection test: qualitative study. JMIR Form Res.

