# Supplementary material for: A Counseling Mobile App to Reduce the Psychosocial Impact of Human Papillomavirus Testing: Formative Research Using a User-Centered Design Approach in a Low-Middle-Income Setting in Argentina
Source: JMIR Form Res. 2022 Jan 13;6(1):e32610. doi: 10.2196/32610 (PMC8796044; doi:10.2196/32610)

Multimedia Appendix 1 – Thematic analysis: Results and Procedures

Content:

- Table 1. KABs about HPV and CC and acceptability and screens evaluation: app's content and features (themes and verbatim).
- Procedures to guarantee trustworthiness criteria.
- Table 2. Citations by themes and groups.
- Images 1-4: Codes distribution by group

©Victoria Sanchez Antelo, Lucila Szwarc, Melisa Paolino, Diana Saimovici, Silvia Massaccesi, Kasisomayajula "Vish" Viswanath, Silvina Arrossi.

Originally published in JMIR Formative Research (https://formative.jmir.org), 06.01.2022. This is an open-access article distributed under the terms of the Creative Commons Attribution License (https://creativecommons.org/licenses/by/4.0/), which permits unrestricted use, distribution, and reproduction in any medium, provided the original work, first published in JMIR Formative Research, is properly cited. The complete bibliographic information, a link to the original publication on https://formative.jmir.org, as well as this copyright and license information must be included

Table 1. KABs about HPV and CC and acceptability and screens evaluation: app's content and features (themes and verbatim).

**Themes**	**Subthemes**	**Verbatim examples**
Knowledge	Lack of information	What is HPV? How did I get it? What happened? What do I have to do?<br>FG3, 30-49 years old
	Partial or incorrect information on HPV transmission	- (...) I was told that HPV couldn't be transmitted in a bathroom or through underwear, that the other thing I told you I had could be transmitted this way, but I was told that HPV could only be transmitted through sexual intercourse, is it true? Not in a public bathroom…<br>-Well, the doctor told me that you could get it in a public restroom, by borrowing clothes, that's what she told me.<br>FG1, 30-49 years old<br><br>Can poor hygiene in a mother's hands transmit HPV to a baby?<br>FG2, aged 50 and over
	Information Needs - Nature of HPV	[HPV] Is it herpes?<br>FG4, aged 50 and over
	Information Needs - Symptomatology	Do you get a fever or sick to your stomach?<br>FG2, aged 50 and over
	Information Needs - Prognosis	Can it be cured?<br>FG1, 30-49 years old
	Information Needs - Who is at risk for HPV	-When you have your first menstruation is... when you turn... at about 18 years old, that's when you get this HPV thing...?<br>FG1, 30-49 years old<br><br>- I had my uterus taken out when I was 36 years old and I had only my cervix. So, I thought I wasn't going to get HPV. (...) I said 'well, that's it, they took out my uterus, I won't get cancer, but I might...' You see, you don't know.<br>FG4, aged 50 and over
	Information Needs - Follow-up medical procedures	- If the app could explain what a biopsy is like. People have it done for the first time and they don't know and can get frightened.<br>- Yes, what it's like, what it's for and why we have to do it. We have to understand... why it's useful. Many of us go, we do it, but we don't know why. We go because we have to go.<br>FG3, 30-49 years old
HPV beliefs	It only affects young people	So, I would tell the girls to get vaccinated and to those who didn't get vaccinated and didn't do things in time... to please get the annual check-ups which are essential because they prevent many issues.<br>FG4, aged 50 and over

	It’s only transmitted in heterosexual intercourse	It’s a sexually transmitted infection that’s transmitted from a man to a woman and may be caused by not having a steady partner.<br>FG2, aged 50 and over
	HPV and cancer as dormant diseases	I've heard that too, that we all have cancer, cancer is asleep until it wakes up or something like that, which I don't know if is true or not, but that's what they say.<br>FG4, aged 50 and over
Attitudes	An HPV+ result cause conflicts with partner - infidelity	In my case, for example, I drove my husband crazy. I was crazy myself because I’m a calm woman who has been by his side all my life, and it's like you feel cheated.<br>FG3, 30-49 years old
	An HPV+ result causes fear of consequences in sexual intercourse	Sometimes you also feel discomfort when you have sex, I was told. The doctor also explained that to me.<br>FG1, 30-49 years old
	An HPV+ result causes concerns on infertility	Concerns [about] fertility, infertility as well.<br>FG1, 30-49 years old
	An HPV+ result causes distress	[On delivery of the result] “It’s to find out if you have cancer”. When [the doctor] said that so coldly, uttering that terrifying disease, I was shocked and then, of course, I went white in the face, pale, I didn't know what to do (...) Then there was another doctor and I asked her: "This was positive, does that mean I have cancer?" "No”, the doctor told me, “who told you that? No, no, this is a probability, this is compared with the Pap smear and with a colposcopy" ...That’s when I calmed down.<br>FG2, 50 years and over
App Acceptability	Trust	- (...) I think the app you’re making is great, it’s very, very, very good...<br>- We’ll be proud knowing that we collaborated, so we’ll be a part of it too…<br>FG2, aged 50 and over<br><br>- [I would use the app] if it's going to help me. I would download it if a doctor recommends it. If the gynecologist tells me: "Look, thanks to all this, we’ve been building an interdisciplinary team with the hospital..."<br>FG3, 30 to 49 years old
Welcome Screen	App Preferences Identity - Gender	- Truth is, I wouldn't care about the gender. I would simply be interested in their words bringing me some peace of mind, in receiving a clear explanation.<br>FG2, aged 50 and over
	App Preferences Identity - Human traits	- I would put, instead of "I’m an app", I would give it a name to generate a more personal bond, you know?<br>- Yes, I think that’s great because the truth is that when... with the greeting "I’m an app" it sounds like a robot and... Yes, you delete an app, I think it would be perfect if they have a human identity, that looks like, simulates, at least pretends to be human.<br>FG3, 30 to 49 years old

Onboarding Screen	Reactions - Confusion	- I don't understand, so you open the app and it says it's going to help you and now it jumps to "what did the doctor say?"<br>-… So, you open the application knowing that you have HPV?<br>-… I think there could be another [screen] before asking "Did you get an HPV test?"<br>FG2, aged 50 and over
Contents Menu Screen	Reactions – Positively evaluated content	- It's great.<br>- I think it's good.<br>- I think it's very good.<br>- I would separate "If you like, I can help you with..." and you have all the information, "Would you like more information?" and then, absolutely, refer you with an appointment... make an appointment to have the study done, no matter what, and to consult a specialist, always.<br>FG4, 50 and over
Information Screen	Reactions – Positively evaluated content	I would read all [the options], because I want to be informed. I want to know. I would put [this screen] first.<br>FG2, 50 and over<br><br>All the [options] they included seem very interesting to me.<br>FG3, 30-49 years old
Myths and Facts Screen (Emotional Support)	Accepts the "Myths and Facts" screen.	- Yes, I'm also very interested in myths because that reduces a lot of stress, because of things that you hear and that you believe to be true.<br>FG3, 30-49 years old
	Rejects the "Myths and Facts" screen	- "HPV easy woman" seems to me... very... I don't know how to put it... these things aren't even related. There are people who think so, but...<br>- Instead of easy woman, promiscuity...<br>- Right, promiscuous.<br>- It's as if you were labeled as an "easy woman", and this is something that...<br>- Sure, maybe you didn't have anything and they brought it [HPV] to you.<br>- Exactly, yes (...)<br>- Of course, it's shocking because of the whole situation we live through. They really are myths, it's true, but when a man reads it and says "easy woman, you're the one to blame", you understand, like....<br>- Or... see how it could be... sexuality, as infidelity, cancer, sexuality alone, there it is... it implies everything, it implies sexual relations, man, woman, sexuality.<br>FG1, 30 to 49 years old
"Things to Make You Feel Better" Screen (Emotional Support)	Preferred Activity - Listening to other women	[Screen "Would It Make You Feel Better If...?"]<br>- Listening to other women who have gone through the same thing is very useful for me.<br>- To tell their experiences, to know how each one of them felt, how they went through it.<br>- Yes, listening to messages from your children or grandchildren, I ... I don't know. I think that, at that very moment, if you're going through something, it's more useful to listen to other women who have

		gone through the same thing because you want to find answers to all the doubts in your mind, in my opinion, my humble opinion.<br>-.... to have a group so that we can talk and interact, tell our testimonies, what each one of us experienced, so that we can exteriorize it.<br>-... that's essential, like what I was saying, the support group, but I agree with what you say about the grandchildren and all that, I don't think it's necessary.<br>- I don't think so either, I agree with you, that it isn't necessary.<br>FG1, 30-49 years old
	Preferred Activity - Sharing with other women	[After reading the screen "Would It Make You Feel Better If...?]<br>-If we're talking about HPV, I would like to listen to stories, and yes, something recorded as a little message, a woman's testimony. Well, regarding the groups, I couldn't agree more. The groups are healing for me, they heal, they help other women with experience so that we can talk about what we know, from the standpoint of how each one went through it, how she faced it.<br>FG4, aged 50 and over
	Dismissed Activities	[Doubtful tone] A list that reminds you of things you like? For what? You always keep them in mind... but listening to or seeing stories from other women, I would like to know that the treatment went well, of course... or information about the group of women who help each other, that would be very useful.<br>FG2, aged 50 and over
"Step by Step – Helpful Information" Screen (Practical support)	Reminder to make an appointment	The appointment reminder is great, super great, amazing, it's fantastic. For people like me who forget things, it's great, I would stop setting alarms for doctor's appointments.<br>FG4, aged 50 and over
	Results availability reminder	And I would include the address of some center or some line where they can call if they've already done the tests and the results were positive and all that. Some number, some hotline or something to be able to talk to someone or maybe even if they haven't gotten the test and they have all the symptoms, to clear that doubt.<br>FG2, aged 50 and over
	Appointments	- Or to make an appointment right there, enter your address and get an appointment at the nearest clinic.<br>FG1, 30-49 years old
	Directory	-What's missing is where to go to receive medical care, "Where can I see a doctor?", and "Where do I go?" ... like a directory.<br>FG4, aged 50 and over
General App Design	Communication Style	[The app] has to use simple words that convey tranquility, that explain things well.<br>FG2, aged 50 and over
	Screen Flow- Contents Organization	- I would like that, before choosing an option on "What did the doctor say?", it would say "Do you know what HPV is?" first. Then if they don't know, send the user to an option where you give her all the information. If she already knows what it is, then follow with the "What did the doctor tell say?" options.

		- I want to find out what [HPV] is all about first, then I want to see, "What method should I follow? Or, if I already know what I should do, "What is [that test] for?".<br>- Also... if you're already in treatment, "What step are you in?", the "step by step" of the process, the appointments and the studies would be missing. If you're already in treatment, "How are you?". Give other options there: "How are you feeling?", "Are you going to a center?", "Are you talking to anyone?" That would also be another bonus point, after the step by step.<br>FG2, 50 and over
	Formats Preferences - Videos	- Well explained videos.<br>- Clear, short but good. Short and educational.<br>- A video of the apparatus...of the ovaries, or of the uterus, what a uterus without HPV would be like and what a uterus with HPV would be like, and so on, explaining...in videos and showing you as well.<br>FG1, 30-49 years old
	Formats Preferences - Multiple formats for accessibility	First you go to the fastest elements, to the illustrations and the videos. Then you go on to the other things, you start reading, but what most draws your attention are the videos and the illustrations. You want to see the symptoms and then you instantly want to see images and then you start reading to understand a little better. But pictures, videos, illustrations are great, and animation too, because maybe you have to explain it to your daughter or to someone who doesn't understand and the animation helps you explain it later, and to understand it yourself.<br>-The text in the video is a big help for people who can't hear, so you also have to think about those people...<br>FG2, aged 50 and over
	Formats Preferences - Images/Photographs	- No pictures, it's very off-putting to see the pictures, today with so much technology... just like the text, we don't need it in the app because we already look for it... it's like it's already there... the video, the illustrations and the animation...<br>- But... I still emphasize awareness, nothing better than a real picture of what can happen.<br>FG4, aged 50 and over

Thematic analysis and procedures to guarantee trustworthiness criteria.

FG audios were transcribed to carry out thematic analysis of the debates [72], based on an iterative and flexible process following six steps: (1) To ensure coding reliability, two researchers become independently familiar with the data through transcriptions and the videorecording. (2) We classified data using an initial codebook based on the theoretical constructs (e.g. knowledge, beliefs, and attitudes) and, in accordance with our research objectives, we identified the reactions to the app and to each draft mock-up screen (opinions); (3) We analyzed each category to generate new themes (e.g. "a salient aspect of the data in a patterned way, regardless of whether that theme captures the majority experience of the participants"[72]). (4) Both researchers met to review themes to identify consistencies and resolved the inconsistencies with the other members of the team. (5) We grouped the emergent themes according to their conceptual similarities to define and name the subthemes, and (6) We sought examples that adequately graphed each theme. Women's preferences, reactions and opinions regarding the app draft screens were coded into emergent categories (subthemes) that identified their preferences in regard to content, tool, feature, and design aspect.

To ensure coding reliability, a third author (SA) verified coding against a sample of transcripts and critically reviewed the data and themes to improve the trustworthiness of the study[75].

Our epistemological assumptions followed Lincoln and Guba's trustworthiness criteria stated to qualitative research: credibility, dependability, confirmability, and transferability [75]. They can be summarized as follows:

- Credibility is to establish the confidence that, from the participants' perspective, the results are true, credible, and believable.

- Dependability implies ensuring the findings of this qualitative inquiry are repeatable if the inquiry occurs within the same coders and context.

- Confirmability requires extending the confidence that the results could be confirmed or corroborated by other researchers.

- Transferability supposes extending the degree to which the results can be generalized or transferred to other contexts or settings.

In order to guaranty the credibility of the data collection and the results, we had the opportunity to engage with each FG participant during the individual interview. This provided us with contextual information about each participant regarding HPV and CC and other relevant characteristics to keep in mind during the FG and therefore enhance our familiarity within the setting. To calibrate the data collection tools, the research team conducted training sessions by teleconferences. Additionally, we conducted a pilot FG with 6 women to test the FG guide in a virtual environment. Through this experience, we evidenced the need to split the guide.

Moreover, the principal investigator verified that the team that conducted data collection had the required knowledge and research skills to perform their roles. We established an Advisory Council to advise on the app design process. The Advisory Council is comprised of prestigious, internationally recognized scientists with extensive expertise in HPV and cervical cancer prevention. We had a debriefing session with the Advisory Council of the project during which we presented the methodological strategy, including the FG guide. Details of the Advisory Council's virtual meeting are available in Spanish at: https://www.cedes.org/diseno-de-una-aplicacion-de-consejeria-para-reducir-el-impacto-psicosocial-del-test-de-vph-un-enfoque-centrado-en-las-usuarias/.

To ensure dependability, the study was conceptualized from the early stages through a systematic review of existing literature (randomized control trials, RCT) that evaluated mobile apps to describe the features and the design process of apps that proved to be effective. We searched for updated research on health-related apps designed in Argentina. We searched at health governmental institutions, non-profit organizations (NPO), other research group websites, and repositories such as PlayStore and AppStore. This review was carried out to identify styles, sources of information available to women, and health information not covered by these apps, yet required by our target population. Additionally, we presented a valuable description of the study methods with detailed steps in data analysis. The progress of the data analysis was monitored weekly and any modifications in the coding

system were discussed and verified to ensure correct and consistent interpretation throughout the analysis.

To guarantee confirmability, we implemented reflexive analysis conducted by the research team who brought different perspectives to the data interpretation. After completing data collection, we sought reflection and feedback from all the researchers conducting the FGs in both written and verbal format.

To assess the confirmability and credibility of the findings, we followed the researchers' triangulation process. Through consensus via team collaboration, discussion and participation, we reached decisions while taking into account various perspectives. We also used the interviewers' field notes and reflexive notes as a form of triangulation to validate the collected data. This approach enabled us to balance out the potential bias of individual investigators and allowed the research team to reach a satisfactory consensus level.

As outlined in the methods section, transferability supposes the use of purposive sampling to ensure that the selected participants were representative of the potential app's end-users. However, this representative aspect was challenging due to the limited number of HPV positive women in the setting. The recruitment was aimed at women with HPV+ results (about 13% of women tested for HPV in our setting). This constitutes an additional recruitment challenge to achieve larger samples. We used age as stratification criteria but, due to the limited number of the target population, we could not include another variable to stratify. Regarding this aspect, we highlight that the qualitative researchers' role is to apply their theoretical expertise to interpret and communicate the diverse perspectives on a given topic.

Lastly, emergent codes were identified in the first FGs followed by a decreasing frequency of codes identified from other FGs. This indicated adequate data saturation to the research objectives.

Table 2. Citations by themes and subthemes.

Color reference	*Themes (codes)*	*Subthemes (subcodes)*	*Citations**
●	Knowledge	No information	5
		Partial or incorrect information on HPV transmission	13
		Information Needs - Nature of HPV	2
		Information Needs – Symptomatology	5
		Information Needs – Prognosis	2
		Information Needs - Who is at risk for HPV	10
		Information Needs - Follow-up medical procedures	11
●	HPV beliefs	It only affects young people	2
		It's only transmitted in heterosexual intercourse	5
		HPV and cancer as dormant diseases	6
●	Attitudes	An HPV+ result cause conflicts with partner - infidelity	3
		An HPV+ result causes concerns on infertility	1
		An HPV+ result causes fear of consequences in sexual intercourse	2
		An HPV+ result causes distress	13
●	App Acceptability	Trust	22
●	Welcome Screen	Reactions-Positively evaluated	2
		App Preferences Identity - Age/Gender	4
		App Preferences Identity - Human traits	11
●	Onboarding Screen	Reactions: Confusion	11
●	Contents Menu Screen	Reactions – Positively evaluated content	4

Information Screen	Reactions – Positively evaluated content	9
Myths and Facts Screen (Emotional Support)	Accepts the "Myths and Facts" screen.	5
	Rejects the "Myths and Facts" screen	9
"Things to Make You Feel Better" Screen (Emotional Support)	Dismissed Activities	6
	Preferred Activity – Listening to other women	7
	Preferred Activity – Sharing with other women	2
"Step by Step – Helpful Information" Screen (Practical support)	Preferred Feature – Appointments	5
	Preferred Feature – Directory, Institutional data (Phone, address)	12
	Preferred Feature – Reminder to make an appointment	4
	Preferred Feature – Results availability reminder	1
	Preferred Feature – Save Medical Information	4
	Preferred Information – How to prepare for follow-up	3
General App Design	Contents Organization – Screen Flow	11
	Contents Presentation	3
	General App Design – Communication Style	5
	Proposed Features – Share app content	8
	Formats Preferences – Illustrations, animations	3
	Formats Preferences – Images/Photographs	8
	Formats Preferences – Multiple formats for accessibility	6
	Formats Preferences – Videos	8
Women presentation		19
**Total citations**		272

*Citations refer to the number of times a coded term was counted in RQDA.

Images 1-4: Codes distribution by group

Image 1. FG1: 6 women, 30-49 years old

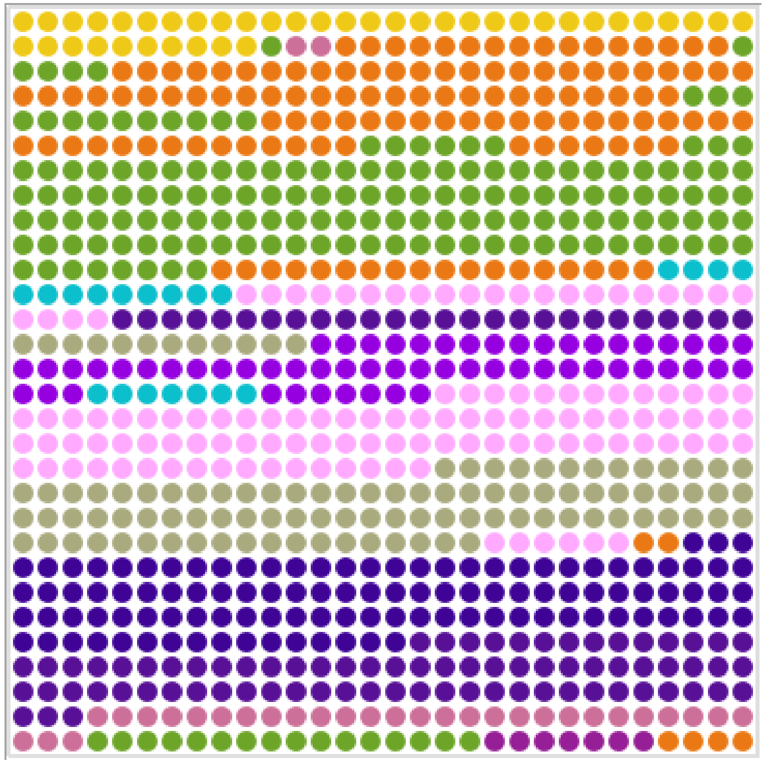

Image 2.FG2: 4 women, 50 and over

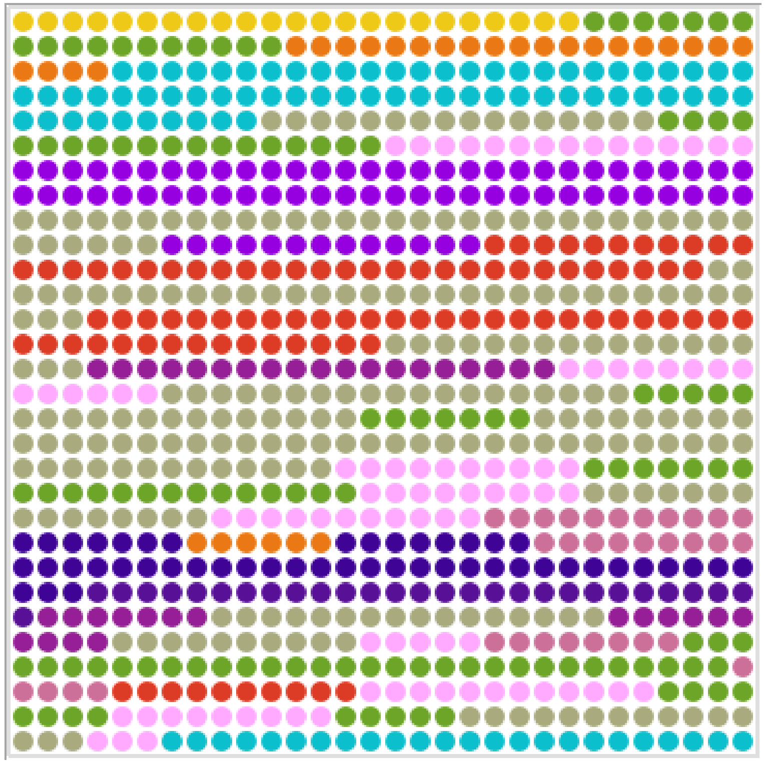

Image 3. FG3: 3 women, 30-49 years old

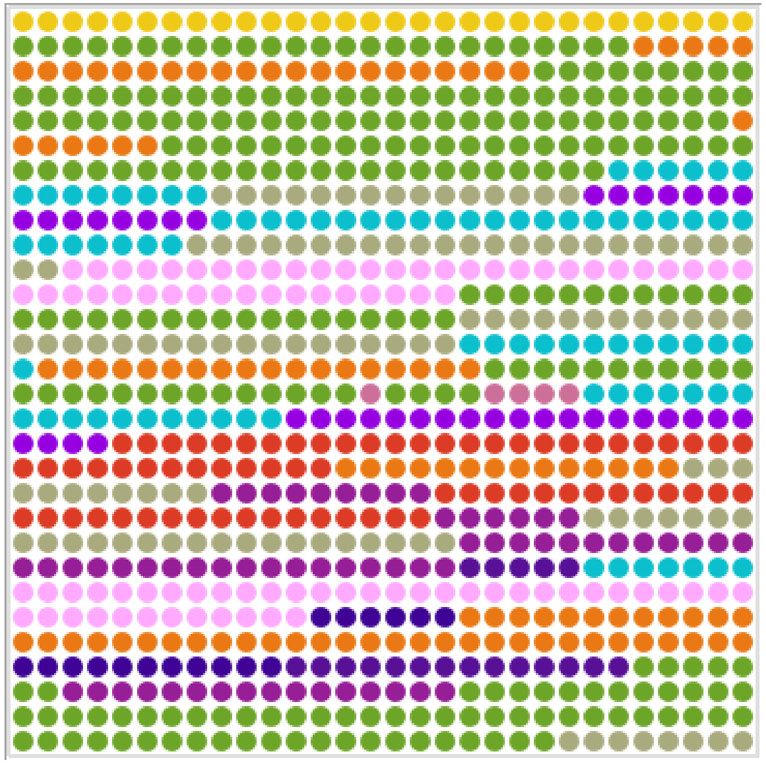

Image 4. FG4: 6 women, 50 and over

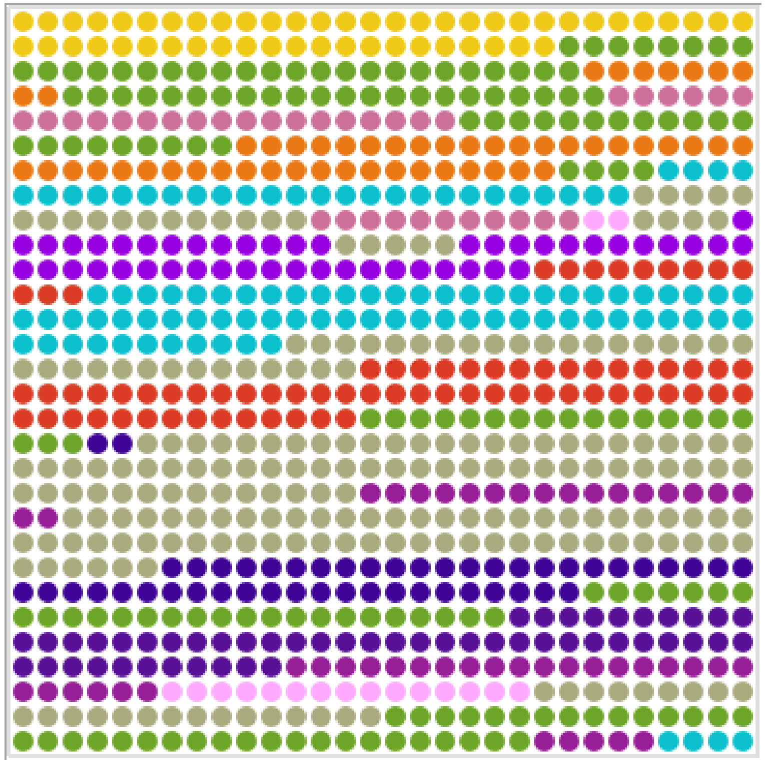

Supplement: Multimedia Appendix 1 [file formative_v6i1e32610_app1.pdf]
